# American Academy of Dental Science

**Published:** 1898-03

**Authors:** 

**Affiliations:** American Academy of Dental Science


### Reports of Society Meetings.

### AMERICAN ACADEMY OF DENTAL SCIENCE.

THE regular monthly meeting of the American Academy of Dental Science was held at Young's Hotel, Wednesday evening, December 1, 1897, at six o'clock, President Cooke in the chair.

A paper was read by Dr. F. B. Lund, entitled "The Place of Local Anæsthesia in Surgery."

(For Dr. Lund's paper, see page 152.)

DISCUSSION.

*Dr. Potter.*—I wish that our essayist would try to use cocaine in some of the cases in which we try to use it. We go into the tough mucous membrane, and underneath that is the periosteum, and every one who has tried it knows that we get into a very hard, unyielding field of operation. Not only that, but when we get an anæsthetized area, we do not always operate there, but at a point somewhat removed, as in the extraction of teeth. Can we reach the nerve-filaments encased in their bony walls? Some of us have done it with success; but I think no one can claim that he always has success. I remember, in 1885, my first use of cocaine by hypodermic injection for the extraction of a tooth. I injected on the outer and inner side of a lower molar root, using a five-per-cent. solution, and putting in at least a minim on each side, and the operation was quite satisfactory. I have used the drug more or less ever since, and it has been my experience that in order to get a very good result in extracting a tooth I need to use a four- or five-per-cent. solution, and put in about one-tenth of a grain. I have searched the authorities very thoroughly to see what is regarded as a therapeutic dose, and I find that one-eighth of a grain is about the maximum for use in regions about the head. It seems to me that in extraction it is necessary to put in about one-tenth of a grain in order to get a satisfactory result. I have had one unfavorable case in the use of this drug, which put me on my guard. We need to have the necessity of care in its use impressed upon us. If we use the very dilute solutions which have been spoken of here to-night, we are not likely to obtain systemic effects; but in my experience the stronger solutions—four and five per cent.—are better for our purpose.

*Dr. Lund.*—I should like to say that I think the region around the teeth and gums would be as unsatisfactory a place to work in as could be found. In other parts of the body one can use of the 1 to 500 solution twenty-five syringefuls, of twenty minims each, in an extensive operation without causing the absorption of a dangerous amount, since a good deal of the injected fluid runs out when the tumors are cut and retracted. Of a 1 to 1000 solution, fifty syringefuls could be used without unduly increasing the amount of cocaine. In the removal of tumors, etc., where there is always more or less extensive dissection, these weak solutions are effectual and really safe. But as to getting inside of the bony channels in the mouth, I should think it would be extremely difficult and uncertain.

*Dr. Piper.*—Will the essayist please tell us how the tissue recovers in those cases where many injections have been made, and what condition it is in after being treated in that way?

*Dr. Lund.*—The fluid is gradually absorbed. It does not interfere with the healing, provided you inject an aseptic fluid. Naturally, if you inject a septic fluid, it suppurates; but if a perfectly clean solution is used, the œdema will disappear after twenty-four hours, or, at least, be displaced by a slight inflammatory swelling without redness, which quickly subsides and does not interfere with healing.

*Dr. Piper.*—I used cocaine in 1885, when it was first introduced into this country, and I have continued its use up to the present time, having used a hydrochlorate solution of from twenty to four per cent. The twenty-per-cent. solution was used on the theory that if a very small amount of a strong solution be injected, a better local anæsthesia would be obtained without alarming systemic symptoms.

The local effect was good, so also was the systemic. I then started to use a ten-per-cent. solution, and the results were very good. I think perhaps I used this solution about five hundred times, without an accident or alarming symptom. Now, however, every patient submits himself to an examination in regard to his physical condition before cocaine is used. Thus, having full knowledge of what may be done and what may be expected, we are prepared to meet any serious obstacle to the success of the operation. One case taught me to take this precaution. The experience of accidents caused by the use of this drug by responsible men, as reported in the leading journals, led me to look about for some other alkaloid to take the place of cocaine hydrochlorate. Stumbling on an article by Dr. Arthur P. Chadbourne on tropacocaine, published in a London journal, I became interested, and immediately procured a few grains, and cautiously began with its use, having the first injection made upon myself for the removal of a small bunch on the side of the face. The operation was painless, without the alarming symptoms which were presented from an injection of hydrochlorate of cocaine some twelve months previous. This was encouraging. I began to use it on patients furnished by a physician, who was in attendance to treat any alarming symptoms that might present themselves. After using tropacocaine some six months, I made an appointment with a surgeon to remove an epithelioma from the face of a patient to whom he did not care to give ether on account of the heart. I suggested the use of tropacocaine. He said, "Very well; if you will take care of the injections, I will take care of the patient." Two grains of the alkaloid were put under the skin before the operation was complete, and, much to our surprise and gratification, not a single symptom from the drug presented itself. While I cannot get the result from tropacocaine that may be secured from cocaine, I prefer to use it because it is much less toxic, and the local anæsthesia very good. I now use tropacocaine more often than I do cocaine.

*Dr. Lund.*—What strength of solution do you use of the tropacocaine?

*Dr. Piper.*—Three per cent. It is more expensive than cocaine.

*Dr. Lund.*—I have tried it only twice, using only a four-per-cent. solution, and I did not get a good result in either instance. The operations in both cases were in inflammatory processes under the lip, and I was discouraged by the failure to produce anæsthesia.

*Dr. E. C. Briggs.*—I want to emphasize what the essayist has called particular attention to, and that is the obtaining of a wheal. After reading Schleich's paper several years ago, I appreciated that point, and I always aim to produce that wheal, and I find, if I get it, the success of the operation is assured. It was with that in mind that the preparation which is put up by Dr. Kitchen, with a petroleum base, caught my fancy, and I have used it now for two or three years. As would suggest itself to you, this petroleum base has the effect of confining the preparation about the point of injection. I find the greatest difficulty is to prevent the effect from going off into the surrounding tissues. Dr. Potter says that he has had difficulty in obtaining the effect in dense tissue. That is just where I can do the best with it. In the softer parts—say, for instance, where the cheek is reflected upon the alveolus—you will find that the cocaine disappears immediately into the loose connective tissues, and you get no wheal, no local anæsthesia, and there is danger of getting constitutional effects. I use cocaine in extracting teeth, injecting next the margin of the gum, producing a wheal; then I make another injection just inside the limits of that wheal, and then another, and by making three injections on each side the tooth can be extracted without pain. Then by running the syringe down into the socket and injecting as near to the apex as you can get, the socket of the tooth can be bored out, for the purpose of replantation or transplantation, and the whole operation can be done without any untoward effect. The day before Thanksgiving I extracted a tooth which was badly affected with pyorrhœa. Although the tooth was loose, it came very hard; and yet in that case the extraction was done painlessly. Then I injected into the root socket and cut out the soft tissue that droops down; unless you do that the tooth does not seem to get down to a good base. Then, as I put on the splint, I remarked to the lady that I was sorry she would not be able to enjoy her Thanksgiving dinner, fearing that her mouth would be sore and she would be unable to use the tooth. When I next saw her she said she was better able to eat on that tooth than at any time previous to the operation.

The drug "eucaine" has been receiving considerable attention lately. I wish to say that, coincident with all the cases in which I have used eucaine, I got indurated spots that did not disappear for a long time. Of course, I have sometimes observed this effect after using cocaine, but in the eucaine it was universal, so I went back to cocaine. I have never had any very serious results with cocaine, except perhaps in the case of a gentleman who apparently died while I was injecting cocaine into the tooth to take out the pulp. I was much alarmed, and injected quantities of brandy, and finally succeeded in reviving him. I often thought of that case with feelings of perturbation when I was using cocaine; at the same time there was always a mental reservation as to the cause of the accident. I did not feel that cocaine was the direct cause, but rather that it was shock. Within a week the same gentleman was in my chair. He had a sensitive cavity, and I was about to use the cataphoric apparatus to benumb the sensitive dentine, but he had no sooner taken the cold, clammy electrode in his left hand than he collapsed. He fell back, turned gray, and presented an entirely different appearance from an ordinary faint. It was an extreme case of shock, and I do not know how to account for it unless he has a very bad tobacco heart.

*Dr. Eames.*—In regard to syncope, I believe that many cases are due to the fact that the patient has eaten nothing for several hours previous to the appointment. Only yesterday I had a case which required some probing in a fistula; it was entirely painless; but the patient immediately fainted. It was about twelve o'clock, and after the patient had recovered I suggested that she should then take lunch, which she did, after which I was able to probe the fistula thoroughly and cut the surrounding tissues without causing syncope in the slightest degree. I have had this experience repeatedly, so that I now do minor operations, not requiring a general anæsthetic, soon after a meal rather than before one.

In regard to the injection of cocaine, I use a syringe having a solid piston, the whole being made of metal, so that it may be boiled or cleansed in any way we wish. For the extraction of a tooth, I believe that it is better to make several small injections on each side rather than to press the needle in deeply and inject the whole quantity at one insertion. By this means we get the "infiltration of tissue" which is desired. With reference to eucaine, I consider it more dangerous than cocaine. I believe that those who declare it to be without danger, do so without knowledge. I will admit that it may be used to a great extent without serious results. It takes very extensive experimentation to arrive at the relative toxic property of such a drug, and we have not had that yet. We know that chloroform may be used hundreds and hundreds of times without fatal results, and yet we know that serious results do occur sufficiently often to influence many of us to abstain from the use of this agent altogether.

*Dr. Brackett.*—I wish to corroborate out of my experience the remarks of the speaker who has just taken his seat in regard to the toxic effects of cocaine solutions being more noticeable in persons who are in need of refreshments. Some of the most unsatisfactory experiences that I have had have been with patients who have come in at an early hour in the morning, having passed a sleepless night, perhaps an interval of several days and nights without sleep, and in many cases having taken but little food. They come to my office in a state of general exhaustion. Patients in that condition are just the ones for whom to be cautious about using cocaine.

I began using the drug as early as it was to be had, have continued its use ever since, and I have felt all along very earnestly that the practitioner who does not avail himself of aid of this kind must be subjecting his patients to a great deal of unnecessary suffering. At first I used strong solutions in considerable quantity. From experience I learned that weak solutions are proportionately more effectual than strong solutions, while, of course, a great deal safer. One of the points which has been brought to my mind this evening is that much more attenuated solutions than those which I have used are effectual. I shall go back to my office and prepare solutions of such strengths as the essayist has indicated and use them with confident expectation of safe and satisfactory results. I feel under especial obligations to the essayist for the information which he has given us.

Early in the use of this drug, Dr. J. W. Smith, who is not now living, put in practice an idea which I believe was original with himself, and which I have previously mentioned before this society.

That was the adding to the solution of a very minute proportion of carbolic acid, which seems to make the cocaine solution less readily absorbable, less likely to produce constitutional effects. From 1889 or 1890 down to the present time it has always been my practice to add a drop of the deliquesced solution of carbolic acid to cocaine solutions, and it is my belief that on account of so doing I have had fewer cases of systemic disturbance. I believe there is another advantage in doing this. Although I always prepare very limited quantities of cocaine solutions at a time, I have felt that the carbolic acid was an additional security against its contracting any septic contamination, and I should, therefore, feel safer in using such preparation in case it had been standing a few days.

With regard to the structure of the tissue into which injections are made, it seems to me that the dentist has some advantages over the operator upon other tissues than the gums. In firm, compact tissues much better results are to be attained than in very loose tissues. An efficient syringe, by means of which considerable force can be supplied, is also important.

*Dr. Williams.*—The essayist made one remark bearing upon an idea with which I have had some experience. He stated that in a case on which he operated, which resulted in collapse, he "hit him over the heart" and started the circulation. I simply wanted to say that at one time chloroform was substituted for ether, and after that chloric ether was substituted for chloroform, and partly from want of knowledge of the proper application of these drugs many accidents resulted. We always gave the anæsthetic gradually, and when cases of apparent collapse came we produced a bellows's motion of the chest and lungs by compressing and relaxing the ribs, and by thus forcing air to the heart made the heart act. Fortunately, we never had a fatal case. I had one patient for whom a few roots were to be taken out, and he suggested that his brother, then a young physician, should administer the anæsthetic. I was very glad to have him, of course, and he came and gave chloric ether,—gave it to him till he thought he was insensible. I then took out the roots, and just as I took out the last one the patient fell back, his face became livid, his lips purple, and his eyes glazed, and his breath ceased. The doctor's face turned as pale as the patient's, and he called for ammonia or some electrical apparatus. I told him there was no time to look for machinery, and that we must keep up the motion of the chest to get the patient breathing. I began at once to press with a steady, intermittent, regular motion. The elastic expansion of the chest drew the air into the lungs and stimulated the heart to keep up its action. I have frequently thought since that if we had lost half a minute in looking for apparatus, it would have been a fatal case. So I believe that in cases of collapse, if you keep up the action of the lungs, you are pretty sure to keep up the action of the heart. Dr. Eames and Dr. Brackett spoke of their unsatisfactory experience with patients early in the morning, and I have had several of those cases. Early in my practice I learned that the morning is the time when the nervous system is most readily exhausted or depressed, and is more easily affected by the use of narcotics or stimulants, and the system is more easily drained by effort, mental or physical, than later in the day. Old Dr. Homans said that he often noticed that a dose of opium early in the morning would have a greater effect than after eleven or twelve o'clock in the day. I remember seeing statistics on the subject of delirium tremens by a French physician, and he stated that all of his cases were morning drinkers,—they took the stimulant at the time when the system was most easily upset. In view of these facts, I have always felt that it was important to save the morning strength. Speaking of that to a lady not long ago, she concurred in my idea, and told me of what she thought was a mistake on the part of one old doctor. He used to get his daughters up in the morning to walk around the Common, and not one of them has any nerves now she said. I have also noticed this in studying the obtunding of sensitive dentine. I have found that patients complained more in the early hours of the day than later in the day, so that I came to adopt the rule to make appointments for what promised to be sensitive cases as late as convenient in the day and not in the morning.

*Dr. Lund.*—I have never used cocaine on the same person more than once on a given day. I think the rule that Dr. Potter spoke of—not using more than one-eighth of a grain—is a very safe one. The face is near the nervous centres, and the effects of the cocaine would be more quickly felt. It would be safe in other parts of the body to go up to half a grain, if you do not do it more than once. I should never go beyond half a grain except in long operations, when a good deal of the cocaine, first injected, has run out through the incision before the remainder is put in.

*Dr. Piper.*—On one occasion I made an injection of a ten-per-cent. solution for the purpose of drilling through the gum for amputation of the end of a root. The operation was successful, without symptoms of cocaine-poisoning. The next day I had occasion to make another injection for the same patient, using the same amount of the solution that I did the day before, and, much to my surprise, certain symptoms presented themselves which have been attributed to a toxic dose of cocaine hydrochlorate. My belief that cocaine may be accumulative is based upon this one case.

*Dr. Eames.*—I should say that cocaine is not cumulative. For the past six years I have used cocaine many times daily, and have carefully observed its effects, and my observations lead me to believe my statement to be correct.

WILLIAM H. POTTER,
*Editor American Academy of Dental Science.*

